# Full use of factors promoting catalytic performance of chitosan supported manganese porphyrin

**DOI:** 10.1038/s41598-020-70210-y

**Published:** 2020-08-24

**Authors:** Lin-Qiang Mo, Xian-Fei Huang, Gao-Cai Wang, Guan Huang, Peng Liu

**Affiliations:** 1 School of Chemistry and Chemical Engineering, Guangxi University, Nanning, 530004 Guangxi China; 2 School of Road and Bridge Engineering, Guangxi Transport Vocational and Technical College, Nanning, 530023 Guangxi China; 3 School of Electrical Engineering, Guangxi University, Nanning, 530004 Guangxi China; 4 School of Computer and Electronic Information, Guangxi University, Nanning, 530004 Guangxi China

**Keywords:** Chemistry, Engineering, Materials science, Mathematics and computing, Nanoscience and technology

## Abstract

In order to make full use of the impact of internal and external factors on the performance of title catalyst for ethyl benzene oxidation, the key internal influencing factors on the catalytic performance were modulated by coordinating and grafting manganese porphyrin to mesoporous and macroporous chitosan, and the important external factors (i.e. oxidation reaction conditions) were optimized using Response Surface Methodology. Under the Response Surface Methodology optimized oxidation reaction conditions (176.56 °C, 0.59 MPa, and 0.25 mg amount of manganese porphyrin), the catalyst could be used at least five times. The ethyl benzene conversion, catalyst turnover numbers, and yields reached up to 51.2%, 4.37 × 10^6^ and 36.4% in average, respectively. Compared with the other optimized oxidation reaction conditions, the corresponding values increased 17%, 26% and 53%. Relative to the manganese porphyrin, the catalytic performance and efficiency of the immobilized catalyst had notably increased.

## Introduction

High-efficiency and high-selectivity oxidation of ethyl benzene (EB) has been still paid a great attention^1–6^. There were so many advanced catalysts and catalytic ethyl benzene oxidation technologies^1–25^, some of which could even offer high yields of products^1–6^.

However, up to date the oxidation techniques used are not very satisfactory yet: There were mainly lack of cleaner production techniques, for examples, O_2_ was not used as oxidant, toxic solvents were used, some energy saving and emission reduction had not been done, and so on. Therefore, the catalyst preparation^21–25^, the catalytic oxidation system and the oxidation conditions^7–20^, which are satisfactorily meeting to the cleaner production process should be comprehensively considered. Based on the above considerations, the internal influencing factors on the catalytic performance of catalyst should be further reasonably tuned, and the external influencing factors should be optimized via Response Surface Methodology (RSM). Because RSM has been successfully used in other catalysis fields: It has been used for optimization of the various reaction conditions and prediction of interactions between the influencing reaction condition parameters based on constructing a suitable model, evaluating the effects of key factors and searching the optimum reaction conditions. The better catalysis responses have achieved^26–29^. These considerations and the various optimizations above will probably bring the advanced catalysis techniques^7–25^ forwards better catalytic results and excellent various applications in practical chemistry industry.

Due to a great deal of attention to the environmental issues, for modern companies which are manufacturing the products of acetophenone and phenylethanol via ethyl benzene oxidation, a set of strict modern technique of cleaner production has to be requested. Under the preconditions, the immobilized metalloporphyrins and their catalysis systems, which are usually not added any additives, co-reductants, and solvents, but oxygen molecules are required as oxidant, will be chosen as one of the excellent catalysis systems adapting to cleaner production. In fact, metalloporphyrin is an excellent catalyst mimicking Cytochrome P450 enzyme. But once-time-exhausted characteristic in catalysis process is one mortal shortage of the unsupported metalloporphyrins. Therefore, the immobilized metalloporphyrins were preferred as a mimicking catalyst relative to the corresponding metalloporphyrins. Because of the concerns of environmental issues, the researches about the catalytic field of the immobilized metalloporphyrins have been greatly promoted and have made a great progress^30^. There were some immobilized metalloporphyrins and their catalysis systems^31–35^ adapting to the cleaner production process. However, the resulting yields (acetophenone and phenylethanol (ONE + OL), less than 25%) were lower than those obtained from the catalytic systems above^7–25^. In addition, RSM was also seldom used in the catalytic field of metalloporphyrins^14–17,19,20,31–35^. Therefore, these are still a challenge.

For promoting the catalytic activity and the re-usability of metalloporphyrins, and increasing the yields (ONE + OL) of ethylbenzene oxidation catalyzed by the immobilized metalloporphyrins catalysis systems, by investigation of the relative references, we found that, (1) the electron-withdrawing-groups metalloporphyrin grafted and coordinated to porous support could comprehensively enhance the catalytic activity and the re-usability for ethylbenzene oxidation^13–15,17,19,20^; (2) the axial coordination action exerted the most important effect on the catalytic activity, and the covalent grafting action had a decisive effect on the increase of the total turnover frequencies (TOFs) and on the re-usability of the catalyst^36^; (3) the most key internal effecting factor on the catalytic activity of the immobilized metalloporphyrin was the axial coordination action^37^; (4) chitosan could be made into nanoporous support material and could provide ammonia groups for the graft and coordination of some metalloporphyrins^34–36^. Especially, it is cheap and no harm to our environment. Therefore, in order to extend our previous related studies^34–36^, manganese (III) 5,10,15,20-tetrakis(pentafluorophenyl)porphyrin (Mn TPFPP) acting as catalytic active sites, has been coordinated and grafted on mesoporous and macroporous chitosan (mCS). In the above way, the mainly internal affecting factors on the catalytic performance of the grafted catalyst have been modulated well, because either the strength of coordination bond or the number of grafted bonds both have been tuned^36^ at this time. Finally, in order to motivate and use external affecting factors on the catalytic performance of the grafted catalyst in a good way, RSM was used for optimizing the external factors, i.e. the ethylbenzene oxidation reaction conditions: Reaction temperature, oxygen pressure and amount of Mn porphyrin. The catalytic performance of the manganese porphyrin coordinated and grafted on mesoporous and macroporous chitosan was studied on ethylbenzene oxidation.

## Experimental section

### Materials

Manganese (III) 5,10,15,20-tetrakis(pentafluorophenyl)porphyrin chloride (Mn TPFPP) , reagents, and chemicals employed were all of analytical grade, and purchased from Sigma-Aldrich in China. Chitosan (CS, MW ~ 7.7 × 10^4^ Da, degree of deacetylation was 90.2%, 4,000 mesh) was purchased from Zhejiang JINKE Biochemistry Co Ltd (China). No oxidation products were found in ethylbenzene by gas chromatography analysis before use.

### Preparation of the supported catalyst (Mn TPFPP/mCS)

22.5 g of chitosan was dissolved in 1,200 mL of acetic acid solution (0.6 mol L^−1^) with stirring for 6 h. The resulting colloidal solution was added dropwise into 10% NaOH solution, forming white chitosan microball. The chitosan microballs were then immersed in a large volume of distilled water for 24 h and washed with deionized water to neutral. The neutral chitosan microballs were added to 45 mL of 25% glutaraldehyde aqueous solution for cross-linking about 24 h, filtered, and then thoroughly washed with deionized water. The resulting chitosan microballs were dried at − 56 °C about 50 h, to obtain mesoporous and macroporous chitosan (mCS) microballs. Under magnetic stirring, 20 g of mCS microballs was added to 800 mL of 1.88 × 10^–5^ mol L^−1^ Mn TPFPP ethylene glycol solution, and the mixture was heated in a microwave oven at 100 °C at least 1 h. The resulting mixture was cooled down to ambient temperature, and filtrated, obtaining mCS-grafted Mn TPFPP microballs. The grafted Mn TPFPP microballs were in turn washed with dichloromethane and ethanol in a Soxhlet apparatus, until no manganese porphyrin in two extracting liquids could be detected using UV–vis spectroscopic method. Hereafter, the microballs were dried at 65 °C under vacuum for 12 h, obtaining Mn TPFPP/mCS catalyst material. Mn TPFPP content in the material was determined by inductively coupled plasma optical emission spectroscopy (ICP-OES) analysis, and which content per gram of the catalyst material was 5.1 × 10^–7^ mol.

**Scheme 1:**
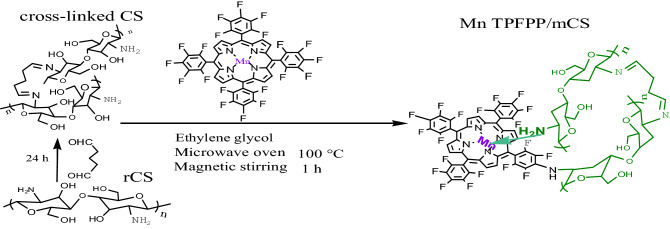
Preparation route for Mn TPFPP/mCS.

When 0.020 g of mCS microballs was added into 500.5 mL of Mn TPFPP ethylene glycol solution (1.88 × 10^–5^ mol L^−1^), in the obtained catalyst material, Mn TPFPP content per gram of the corresponding catalyst material was 4.8 × 10^–7^ mol. The preparation outline of Mn TPFPP/mCS shows as following Scheme 1.

### Measurement

Inductively coupled plasma optical emission spectrometry (ICP-OES, Optima 2100DV) was used for measuring the manganese porphyrin content^36^. Scanning electron microscopy (SEM) was performed in a Hitachi S-3400 N microscope^33,36^. Scanning transmission electron microscopy (STEM) was conducted on a TF20 at an operating voltage of 200 kV^33,36^. The BET surface areas and porosity were measured by N_2_ adsorption at − 196 °C on a TriStar II 3,020 sorptometer^36^. UV–Vis spectra were collected on a Shimadzu UV-1800 spectrometer^38,39^. Infrared (IR) spectra were measured on an iS50 Nicolet spectrophotometer with a resolution of 4 cm^−1^^40–43^. X-ray photoelectron spectroscopy (XPS) was performed on a Kratos Ultra Axis DLD spectrometer with the Al K_α_ as an exciting source at 40 eV^44–46^. Energy dispersive spectra (EDS) were conducted on a S-3400 N scanning electron microscope coupled with an energy dispersive analysis detector^33^. Thermogravimetric and differential scanning calorimetry (TG-DSC) was conducted on a STA 409 PC/PG Luxx thermo analyzer in an atmosphere of air with a flow rate of 20 mL per min^35^.

### Ethyl benzene oxidation and test

Ethyl benzene oxidation catalyzed by Mn TPFPP/mCS was performed according to an optimization experiment design of Response Surface Methodology. A Design-Expert Software (version 8.0.5b) was used to study the experimental design, statistical analysis, and regression model. The model is as following:

1 $$Z = \lambda_{0} + \sum\limits_{j = 1}^{n} {\lambda_{j} } X_{j} + \sum\limits_{j = 1}^{n} {\lambda_{jj} } X_{j}^{2} + \sum\limits_{j = 1,i = 1}^{n} {\lambda_{ji} } X_{j} X_{i}$$

The quadratic Eq. (1) is come from RSM, the symbol Z indicates a predicted response value, which stands for turnover number (TON) of Mn TPFPP/mCS catalyst. X_j_ are independent variables (reaction temperature, reaction pressure, and amount of Mn TPFPP), X_j_^2^ indicate quadratic parameters, X_j_X_i_ mean interaction parameters. λ_0_ is the regression coefficient for intercept term, λ_j_, λ_jj_, and λ_ji_ are corresponding regression coefficients. The "n" represents the number of the independent variables which is 3 in our paper, so we have the following Eq. (1):

2 $$Z = \lambda_{0} + \sum\limits_{j = 1}^{3} {\lambda_{j} } X_{j} + \sum\limits_{j = 1}^{3} {\lambda_{jj} } X_{j}^{2} + \sum\limits_{j = 1,i = 1}^{3} {\lambda_{ji} } X_{j} X_{i}$$

The independent and interactional effects of three factors (reaction temperature (X_1_), reaction pressure (X_2_), and amount of catalyst (X_3_)) on catalyst turnover number were evaluated via a three-level-three-factor experimental design. The independent variables coded at three level (− 1, 0, and + 1) and their change ranges were listed in Table 1. The total number of experiments for 17 listed in Table 2 were obtained according to a 3^3^ Box–Behnken experimental design (BBD).

**Table 1:** Experimental range and levels for Box–Behnken design.

Variables	Range and level		
	− 1	0	1
Reaction temperature (X_1_)/°C	170	175	180
Reaction pressure (X_2_)/MPa	0.50	0.60	0.70
Amount of catalyst (X_3_)/mg^#^	0.25	0.50	0.75

^a^Amount of catalyst = amount of Mn TPFPP.

**Table 2:** Response surface design and experimental design.

Standard	Temperature/°C	Pressure/MPa	Amount of Mn TPFPP/mg	Experimental TON × 10^4^	Predicted TON × 10^4^
1	170	0.5	0.5	114.89	114.8836
2	180	0.5	0.5	105.62	105.7017
3	170	0.7	0.5	90.15	89.76126
4	180	0.7	0.5	98.05	97.7334
5	170	0.6	0.25	199.63	199.0837
6	180	0.6	0.5	116.87	116.5626
7	170	0.6	0.5	116.25	117.1674
8	180	0.6	0.75	46.07	46.60729
9	175	0.5	0.5	127.48	127.3777
10	175	0.6	0.5	134.56	133.95
11	175	0.6	0.75	80.36	79.81871
12	175	0.7	0.5	110.12	110.8323
13	175	0.6	0.25	231.98	231.6903
14	175	0.6	0.25	231.87	231.6903
15	175	0.6	0.25	231.67	231.6903
16	175	0.6	0.25	230.87	231.6903
17	175	0.6	0.25	231.52	231.6903

**Scheme 2:**
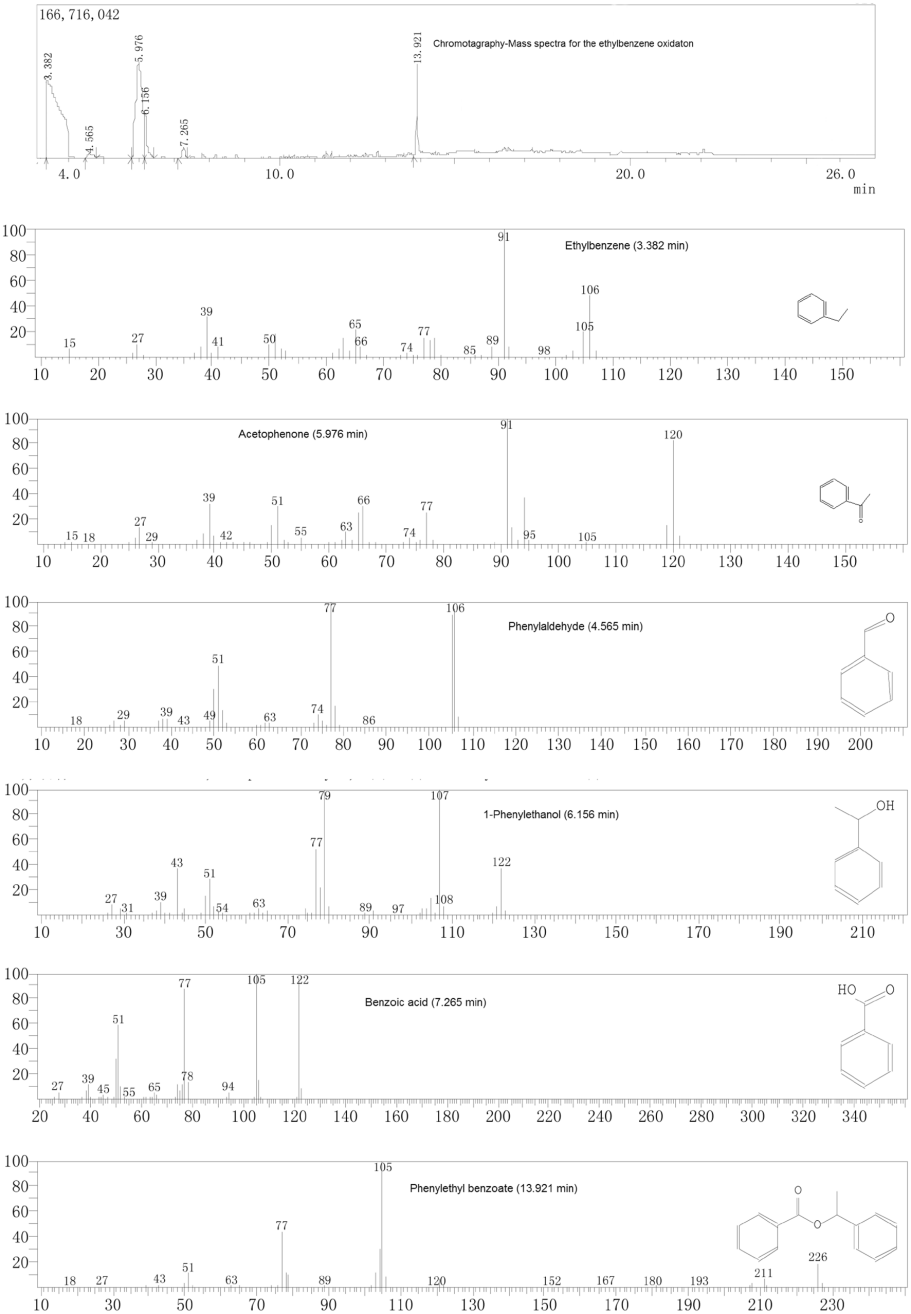
Chromatography-Mass spectra for the ethylbenzene oxidation.

**Scheme 3:**
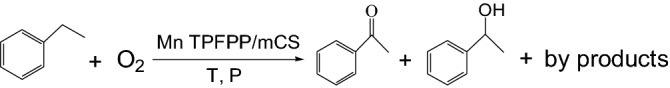
Ethyl benzene oxidation catalyzed by Mn TPFPP/mCS.

Ethyl benzene oxidation catalyzed by Mn TPFPP/mCS was conducted under 0.1 MPa of N_2_, in an autoclave reactor equipped with a magnetic stirrer, a frozen ethanol re-condenser at − 20 °C, and a tail gas flow meter. The reaction mixture was stirred and heated to the designed reaction temperature in Table 2_._ To achieve a designed reaction pressure, O_2_ was continuously introduced into the reaction system. The flow rate of tail gas was kept at 0.03 m^3^/h. The catalyst amount of Mn TPFPP was used in the range of 0.25–0.75 mg. Generally, the time of the catalytic ethyl benzene oxidation was 2.5 h and ended, because after 2.5 h of reaction time, TON and yields did almost not increase again under the reaction conditions of the experimental Design above. During the periods, liquid samples of the oxidation mixture withdrawn every 30 min were qualified by Gas Chromatography–Mass Spectrometry (GC–MS, as Scheme 2), and were quantified the contents of acetophenone, phenylethanol, and phenylaldehyde. The quantifi-cation operation was conducted on the Shimadzu GC-16A chromatograph equipped with a 30 m × 0.32 mm × 0.5 µm FFAP capillary column and a flame ionization detector, using bromobenzene as the internal standard reference. The amounts of by-products, benzoic acid and phenylethyl benzoate were determined using acid–base titration, with phenolphthalein and methyl orange as indicators respectively. The grafted catalyst was then separated from the oxidation mixture by filtration, washed with alcohol to remove residual oxidation products, air-dried, and reused in next ethyl benzene oxidation. The ethyl benzene oxidation reaction catalyzed by Mn TPFPP/mCS is showed in Scheme 3.

The catalyst turnover number (TON), ethyl benzene conversion, selectivity, yields, and relative product concentrations (denoted as C_i_) are defined as followings:

3 $${\text{TON}} = \frac{{{\text{Moles}}\;{\text{of}}\;{\text{total}}\;{\text{products}}\;{\text{formed}}}}{{{\text{Moles}}\;{\text{of}}\;{\text{Mn}}\;{\text{in}}\;{\text{catalyst}}}}$$

4 $${\text{EB}}\;{\text{conversion}} = \frac{{{\text{Moles}}\;{\text{of}}\;{\text{total}}\;{\text{products}}\;{\text{formed}}}}{{{\text{Moles}}\;{\text{of}}\;{\text{EB}}}}$$

5 $${\text{Selectivity}} = \frac{{{\text{Moles}}\;{\text{of}}\;{\text{products}}\;({\text{ONE}} + {\text{OL}})\;{\text{formed}}}}{{{\text{Moles}}\;{\text{of}}\;{{\text{total}}}\;{\text{products}}\;{\text{formed}}}}$$

6 $${\text{Yields}}\;({\text{ONE}} + {\text{OL}}) = \frac{{{\text{Moles}}\;{\text{of}}\;{\text{products}}\;({\text{ONE}} + {\text{OL}})\;{\text{formed}}}}{{{\text{Moles}}\;{\text{of}}\;{\text{EB}}}}$$

7 $${\text{C}}_{{\text{i}}} = \frac{{{\text{(Mole}}\;{\text{of}}\;{\text{product}}\;{\text{formed)}}}}{{({\text{unreacted}}\;{\text{EB}} + {\text{total}}\;{\text{products}})\;{\text{volume}}}}$$

In which, EB conversion is ethyl benzene conversion, Yields (ONE + OL) means the yields of acetophenone and 1-phenylethanol, i stands for acetophenone (ONE),1-phenylethanol (OL), benzaldehyde (BA), benzoic acid (BzA), or phenylethyl benzoate (PEB).

## Results and discussion

### Characterization of materials

**Figure 1:**
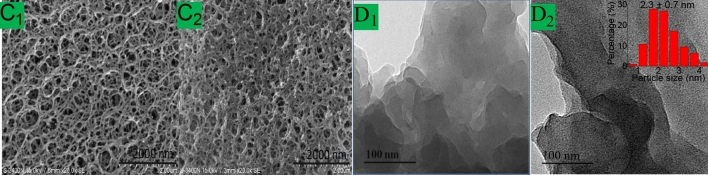
SEM and STEM images of samples mCS (C_1_ and D_1_), and Mn TPFPP/mCS (C_2_ and D_2_).

The morphology and the micro-structure of the as-prepared materials were investigated by SEM and STEM techniques. In the SEM images (Fig. 1C1 and C2), mCS and Mn TPFPP/mCS both showed an porous texture characteristics in morphology. However, there were obvious differences between them. The structure of Mn TPFPP/mCS showed a more dense texture-threads relative to that of mCS, which was probably caused by covalent-bonding of Mn TPFPP molecules to mCS (C_2_ vs C_1_). Further, according to the STEM micro-graphs, there were many little size black spots in Mn TPFPP/mCS (Fig. 1D2), but no any those black spots in mCS (Fig. 1 D1). When the diameter sizes of the black spots in Fig. 1 D2 are statistically calculated with ImageJ software, the black spot sizes are approximately 2.3 nm in average, indicating the highly dispersion of Mn TPFPP molecules (diameter = 1.75^47^) on surface of mCS. This means that Mn TPFPPs have been immobilized and highly scattered on mCS, forming catalytic active sites.

**Figure 2:**
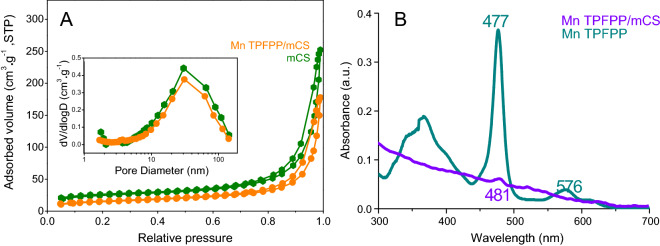
(**A**) Nitrogen adsorption–desorption isotherms for the mCS (a) and the Mn TPFPP/mCS (b). Inset shows the pore size distributions. (**B**) UV–Vis spectra of dichloromethane solution of Mn TPFPP, and dichloromethane suspension of Mn TPFPP/mCS.

Figure 2A presents the experimental results of N_2_ adsorption and desorption for the mCS (S_BET_ = 40 m^2^/g, pore diameter = 21 nm) and the Mn TPFPP/mCS (S_BET_ = 59 m^2^/g, pore diameter = 20 nm), respectively. The results show that these materials mainly possessed mesoporous and macroporous structure characteristics^33^, with an average pore-diameter approximately 20 nm. This is because the BET surface areas of two materials were too small, and the hysteresis loops of isotherms at high relative pressure were due to interparticle volume not to mesopores. Such abundant mesopores existing in Mn TPFPP/mCS will be beneficial for contacting reactants to active sites, catalytically oxidizing substrate, and diffusing products. This means that, one (i.e. porous cavity) of three internal factors had been modulated.

**Figure 3:**
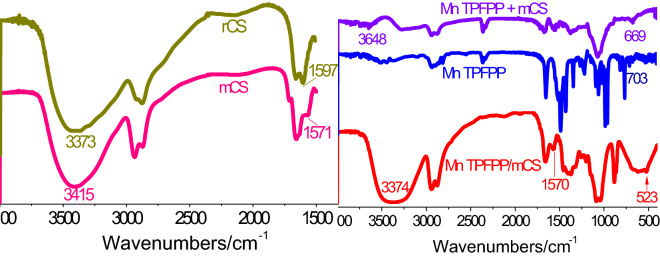
FT-IR spectra of rCS, mCS, Mn TPFPP, mCS + Mn TPFPP, and Mn TPFPP/mCS.

The UV–Vis spectra of the unsupported and supported Mn TPFPP catalysts are displayed in Fig. 2B. Compared to Mn TPFPP, the Soret band in Mn TPFPP/mCS red-shifted 4 nm. This is because that, the lone-pair electron of nitrogen of amino in mCS was axially coordinated to metalloporphyrin to augment its electron cloud density, increasing the a_2μ_(π) energy, decreasing the energy gap between a_2μ_(π) and e_g_(π*) , and leading to the lessening of the needed excited energy^38,39^. Therefore, the Soret peak red-shift indicates there was a coordination bond between Mn TPFPP and mCS. This can be further proved by the relative changes of the following IR spectra (Fig. 3). In the IR spectrum of raw chitosan (rCS), the peaks at 3,373 and 1597 cm^−1^ are ascribed to the absorption bands of –NH_2_ group and amide II, respectively^40,41^. However, the –NH_2_ absorption bands in IR spectrum of mCS shifted to near 3,415 cm^−1^, and the amide II bands shifted to 1571 cm^−1^. These are because the partial amino groups of rCS were cross-linked with aldehyde groups in glutaraldehyde, forming of imine bond (C=N). The cross-linking action resulted in a reduction of amino group numbers in mCS. When Mn TPFPP was supported onto mCS, forming Mn TPFPP/mCS, two interesting changes could be found: (1) The original band of the amino groups at near 3,415 cm^−1^ in IR spectrum of mCS red-shifted to the corresponding band at 3,374 cm^−1^. (2) The original band of amide II at 1571 cm^−1^ was changed into a weaker band at 1,570 cm^−1^. Resulting in the two changes are because the para-F on benzene ring in Mn TPFPP was nucleophilically substituted by amino group (–NH_2_) of mCS^42^. However, there were not the similar changes of spectroscopic characteristics for the mechanic mixture of Mn TPFPP with mCS. This indicates that Mn TPFPP has been covalently bonded to mCS. In addition, a new peak appearing at 523 cm^−1^ for Mn TPFPP/mCS was attributed to the formation of Mn–N bond. This shows that there was an axial coordination bond between Mn TPFPP and mCS^43^. From the above experimental results, we can see that, when the mol ratios of amino group to Mn porphyrin were decreased from 1,237 (1.86 × 10^–2^/1.504 × 10^–5^) to 2 (1.86 × 10^–5^/9.41 × 10^–6^), the Mn TPFPP contents per gram of the catalyst material were very closed, being 5.1 × 10^–7^ mol and 4.8 × 10^–7^ mol, respectively. This indicates that, all added Mn TPFPP molecules were completely coordinated and grafted on mCS.

**Figure 4:**
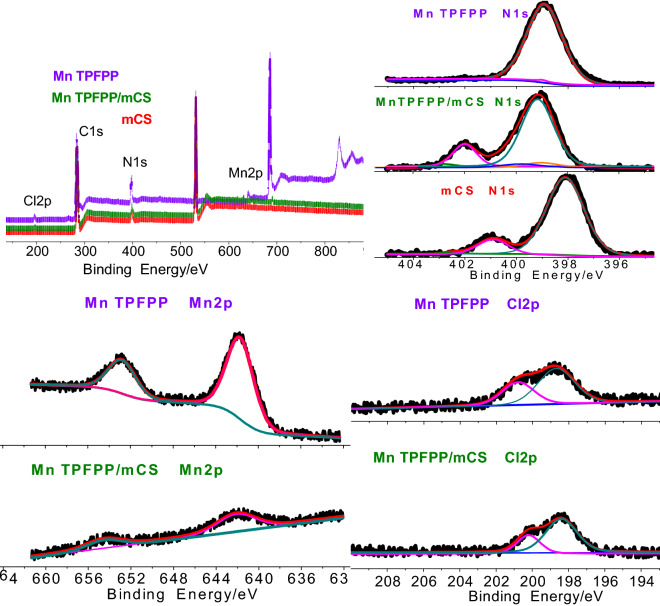
XPS spectra of Mn 2p, N 1 s and Cl 2p for mCS, Mn TPFPP, and Mn TPFPP/mCS.

After covalently bonding and axially coordinating of Mn TPFPP to mCS, the binding energies (BEs) of all elements, especially those of the C, N, Cl and Mn, were changed in different degrees (Fig. 4 and Table 3)^44–46^. The C 1 s binding energy (BE) for the C=C–Funit in Mn TPFPP was changed from 287.0 eV to 284.8 eV for that of Mn TPFPP/mCS. This is because the para F atom of benzene ring in Mn TPFPP was nucleophilically substituted by the amino in mCS, forming Mn TPFPP/mCS via C–N bond. The BE (398.1 eV) of N_1s_ for NH_2_–C_sp_^3^ unit in mCS shifted to the BE (399.0 eV) of N_1s_ for NH_2_–C_sp_^3^ unit in Mn TPFPP/mCS. It means that, the Mn ion of Mn TPFPP was axially coordinated by mCS. These grafting and coordinating actions resulted in the BE decrement (dBE: − 0.6 eV and − 0.3 eV) of Cl 2p in Mn TPFPP. More important is that, the two actions resulted in the BE increment (dBE: 1.7 eV and 0.4 eV) of Mn ion in Mn TPFPP/mCS. This means that the Mn ion of Mn TPFPP/mCS carried more positive charges to bind and activate oxygen molecules, exhibiting its higher catalytic activity than before^48^.

**Table 3:** Electron Binding energy for key elements in mCS, Mn TPFPP and Mn TPFPP/mCS.

XPS Spectra	Element form	Binding energy/eV			
		Mn TPFPP/mCS	Mn TPFPP	mCS	dBE/eV
C1s	C=*C*–F	285.0	287.0	–	–
	C=*C*–NH	284.8	–	–	− 2.2
N1s	*N*–C=	399.7	399.0	–	0.7
	_equatorial_*N*–Mn	399.3	398.9	–	0.4
	*N*H_2_–C_sp3_	399.0	–	398.1	0.9
Mn 2p	*Mn*–N	654.6	652.9	–	1.7
		642.1	641.7	–	0.4
Cl 2p	*Cl*-Mn	200.2	200.8	–	− 0.6
		198.4	198.7	–	− 0.3

Summarily, after grafting and coordinating Mn TPFPP to mCS, the other two of three internal influencing factors on the catalytic performance of Mn TPFPP/mCS had been modulated well. Because the grafting and the coordination determined the recycle use and the catalytic activity of Mn TPFPP, the coordination and the grafting enhanced the catalytic efficiency and activity of Mn TPFPP/mCS. In the same time, it was avoided that too much internal influencing factors will disturb the RSM-designed experimental test.

### The optimization of external influencing factors on the catalytic activity of Mn TPFPP/mCS by RSM

In fact, there were many factors of impacting the catalytic performance of Mn TPFPP/mCS. As far as we know, there were still volume of ethyl benzene, reaction time, stirring speed, tail gas flow rate and solvents (belong to external factors), besides the internal factors and the external factors described above. For avoiding the disturbances coming from too many effecting factors, first, the above internal factors were modulated by the grafting and coordinating Mn TPFPP on mCS^36^. Second, the just-described external factors, were made to be constant, i.e. those were set as 200 mL, 2.5 h, 200 rpm, 0.03 m^3^/h and no any solvents, respectively (Fig. S1-(1 to 4)). But reaction temperature, O_2_ pressure and catalyst amount were acted as variables. All these designs, including the data set in Tables 1 and 2, which were screened from the data (Fig. S1-(1 to 4) obtained via Other Optimization Method (OOM)^34,35^), were set up based on preliminary experimental test data. Especially, the key effects of the external factors (reaction temperature and pressure) on catalyst turnover number (TON) were remarkably occurred at the ranges of 170–180 °C and 0.5–0.7 MPa respectively. In the same time, the specifically key effect of Mn TPFPP amount was acted at the range of 0.75 mg to infinitesimal [this is decided by Eq. (3)]. Further, the precise values of optimal oxidation reaction conditions were investigated using RSM. In a word, after fully utilizing the superiority of internal factors about grafting and coordinating of Mn TPFPP to mCS, further, the external factors, such as reaction temperature, O_2_ pressure and catalyst amount were optimized via RSM.

**Figure 5:**
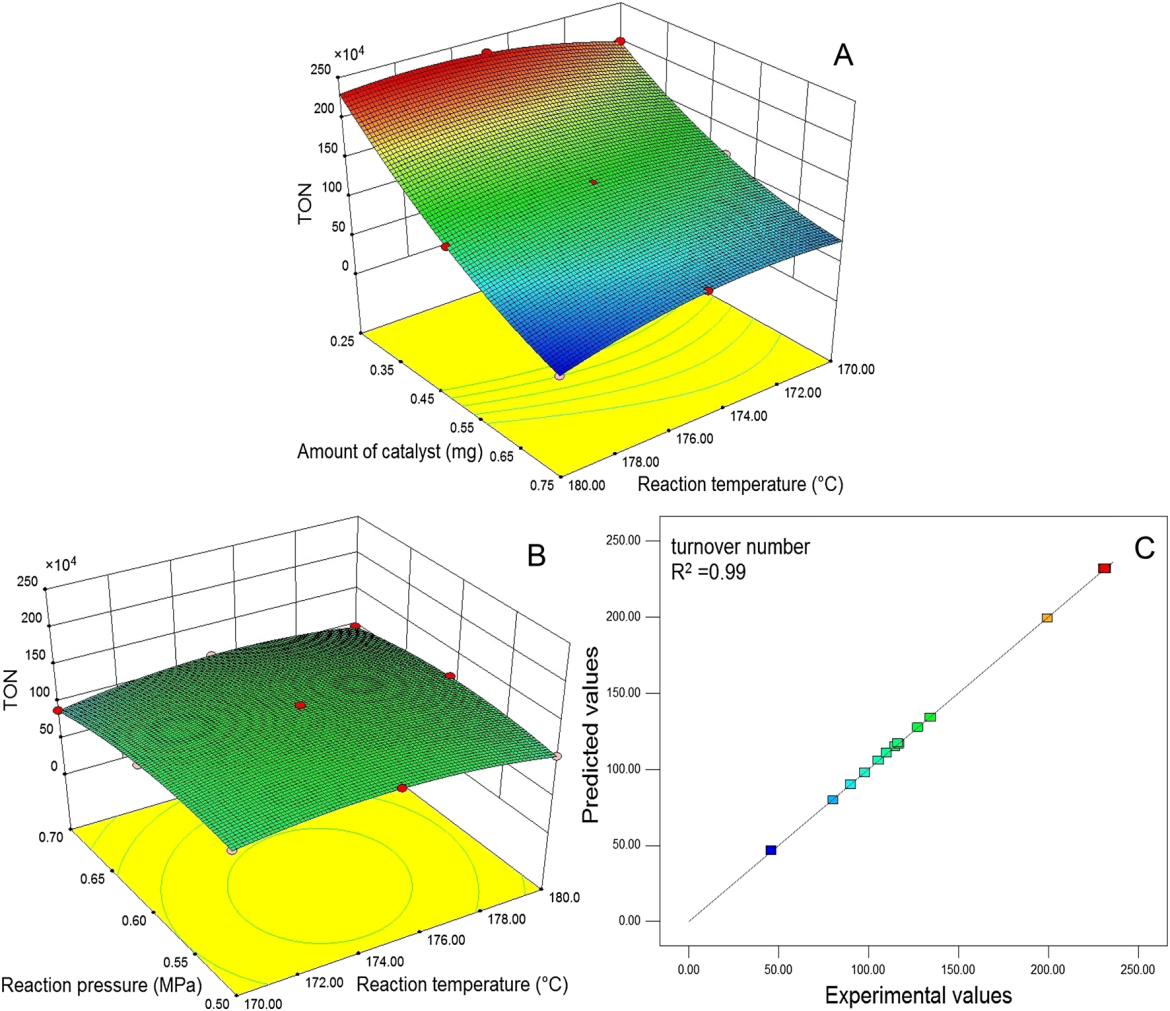
Three dimensional response surface and contour plots for effects of the interactions between reaction temperature and amount of catalyst (**A**), and reaction temperature and reaction pressure (**B**); relationship between predicted value and experimental value plot of the model (**C**).

The experimental data (parameters and experimental TON) in Table 2 was obtained according to the optimization experimental design and the related experiments. These data could be analyzed by the second order polynomial Eq. (3) and the analysis of variance (ANOVA). Subsequently, the predicted TON in Table 2 and the correspondingly reliable regression coefficients in Eq. (4), were obtained,

4 $${\text{TON }} = + {133}.{95} - 0.{3}0{\text{X}}_{{1}} - {8}.{\text{27X}}_{{2}} - {75}.{\text{94X}}_{{3}} + {4}.{\text{29X}}_{{1}} {\text{X}}_{{2}} - {15}.{\text{82X}}_{{1}} {\text{X}}_{{3}} - {17}.0{\text{8X}}_{{1}}^{{2}} - {14}.{\text{84X}}_{{2}}^{{2}} + {21}.{8}0{\text{X}}_{{3}}^{{2}} ,$$

in the same time, some three-dimensional (3D) response surface plots (Fig. 5) and Table 4 were obtained as well. The Eq. (4) stood for the regression model for the variables, such as reaction temperature, reaction pressure, and amount of Mn TPFPP. This Eq. (4) shows that, single variables (X_1_, X_2_ , X_3_ ), their interactions (X_1_X_2_, X_1_X_3_, X_2_X_3_) and quadratic parameters (X_1_^2^, X_2_^2^, X_3_^2^) exerted differently effects on TON. To have a more intuitive understanding the acting effects of interaction parameters on TON, the 2 plots in Fig. 5A,B were plotted respectively by maintaining at the zero levels (0.6 MPa and 0.50 mg, respectively)^49^. Then, some standards in Table 4 offered by BBD are used to illustrate the meaning of those data values in Table 4, including whether or not those parameter terms being significant to the model (Eq. 4) and so on (see followings).

**Table 4:** ANOVA results of the quadratic model for catalytic oxidation of ethyl benzene.

Sources of variation	Sum of squares	Degrees of freedom	Mean square	F-value	P-value Prob > F
Model	65,287.2	8	8,160.90	17,236.8	< 0.0001^a^
X_1_-temperature	0.66	1	0.66	1.40	0.2706^b^
X_2_-pressure	410.62	1	410.62	867.28	< 0.0001^a^
X_3_-amount of catalyst	27,870.17	1	27,870.17	58,865.12	< 0.0001^a^
X_1_X_2_	73.56	1	73.56	155.38	< 0.0001^a^
X_1_X_3_	198.95	1	198.95	420.20	< 0.0001^a^
X_1_^2^	583.79	1	583.79	1,233.04	< 0.0001^a^
X_2_^2^	440.75	1	440.75	930.91	< 0.0001^a^
X_3_^2^	583.95	1	583.95	1,233.37	< 0.0001^a^
Residual	3.79	8	0.47		
Lack of Fit	3.03	4	0.76	3.98	0.1046^b^
Pure Error	0.76	4	0.19		
Cor Total	65,290.99	16			
Std. Dev	R-squared		C.V %	Adj R-squared	Adequacy precision
0.69	0.99		0.47	0.99	369.682

^a^Significant.

^b^Insignificant.

From Eq. (4) and Table 4, we can see that, the effect of Mn TPFPP amount (X_3_) on TON was the greatest among the three single variables in Eq. (4). Because the regression coefficients were obtained from the viewpoint of mathematical statistics^49^. For the regression coefficient of X_3_ term, at its zero level (175 °C and 0.60 MPa), TON first increased and then decreased with increasing Mn TPFPP amount from 0.25 to 0.75 mg. The statistic negative effect was far bigger than the corresponding positive effect, showing an antagonism. Compared with the two antagonisms of X_2_ and X_1_ terms at their corresponding zero levels, the antagonism of X_3_ term was the biggest (coefficient value = 75.94), next was that (coefficient value = − 8.27) of X_2_ term. Therefore, the effect of Mn TPFPP amount (X_3_) on TON was most significant (F-value, 58,865.12 in Table 4).

Figure 5 shows the effects of the interaction parameters on TON. As for as the interaction (X_1_X_3_) between Mn TPFPP amount (X_3_) and reaction temperature (X_1_) is concerned (Fig. 5A), when the oxygen pressure reached at its zero level (0.60 MPa), considering each given reaction temperatures, the micro changes of TON in increasing catalyst amount from 0.25 mg to 0.75 mg, were always negative effect, no any positive effect. Obviously, the statistic negative effect was very large. In the meantime, considering each given Mn TPFPP amount, the micro changes of TON in increasing reaction temperature from 170 °C to 180 °C, showed almost equal statistic negative and positive effects. Therefore, the comprehensive statistic effect of the situations above was negative (coefficient value = − 15.82). It means that, the mutual interaction (X_1_X_3_) had an antagonistic effect on TON and a F-value of 420.20 (Table 4), showing a greater significant effect than that of the mutual interaction (X_1_X_2_). As for as the interaction (X_1_X_2_) between reaction temperature (X_1_) and reaction pressure (X_2_) is concerned (Fig. 5B). When the catalyst amount reached at its zero level (0.50 mg), considering each given reaction temperatures, the micro changes of TON in increasing reaction pressure from 0.50 to 0.70 MPa, the statistic positive effect was slightly bigger than the corresponding negative effect. In the same time, considering each given reaction pressure, the micro changes of TON in increasing reaction temperature from 170 to 180 °C, the statistic positive effect was also slightly bigger than the corresponding negative effect. The net results for both situations showed a little synergistic effect (coefficient value =  + 4.29). It means that, the mutual interaction (X_1_X_2_) had a synergic effect on TON, and a F-value of 155.38, presenting a slight significant effect on TON.

As far as the effects of quadratic parameters (X_1_^2^, X_2_^2^ and X_3_^2^) on TON are concerned, the coefficient value of the quadratic parameter (X_3_^2^) was + 21.80 [Eq. (4)], showing a positive effect of X_3_^2^ on TON, displaying a greatest synergic effect (synergy), and having a greatest significant effect. Because the F-value was 1,233.37 (Table 4). For the effect of quadratic parameter (X_1_^2^), the coefficient value was—17.08, showing a negative effect of X_1_^2^ on TON, displaying a greater antagonism, and having a bigger significant effect. Since the corresponding F-value was 1,233.04. Obviously, X_2_^2^ showed the smallest negative effect on TON, also the least significant.

In summary, among all type parameter terms, the single parameter (X_3_) and the quadratic parameter (X_3_^2^ and X_1_^2^) showed the most significant effect on the catalytic activity (TON). Next were the quadratic parameter (X_2_^2^), the single parameter (X_2_), and the mutual interaction (X_1_X_3_). The last was the interaction (X_1_X_2_).

Therefore, under the intricately oxidation conditions involving in multiple reaction parameters, when it is lack of RSM, seeking for the best and precise ethyl benzene oxidation reaction conditions is already very difficult. However, with the help of RSM, it has been easily obtained and the such best and precise oxidation reaction conditions are as followings: X_1_ being 176.56 °C, X_2_ being 0.59 MPa, and X_3_ being 0.25 mg Mn TPFPP. These data can be also found in Fig. 5A, B.

For evaluating the accuracy of the RSM used, the BBD had also offered Fig. 5C. From which we can see that, either the actual values or the predicted ones (for TON values), both were well distributed very close to a straight line, and R^2^ was 0.99. This indicates that the RSM used was accurate enough.

There are 6 types of other important data in Table 4, being as followings: P-value, Lack of Fit, R-squared, C.V %, Adj R-squared, and Adequacy precision. When the *P* value was less than 0.0001, the corresponding parameter term was significant; but if it were more than 0.1, the parameter term would be insignificant. Because the Adequate precision was more than 4, this signal to noise ratio could be acceptable, and this model could navigate the design space. The value (0.47) of the coefficient of variation (C.V %) indicates that, the reliability of simulated experiments by RSM, and its model accuracy were well. The Eq. (4) was effective because the value of lack of fit was more than 0.05. The R^2^ value equaled that of the adjusted R^2^, which was close to 1. This means that, the RSM simulation to the present experimental data was accurate.

**Figure 6:**
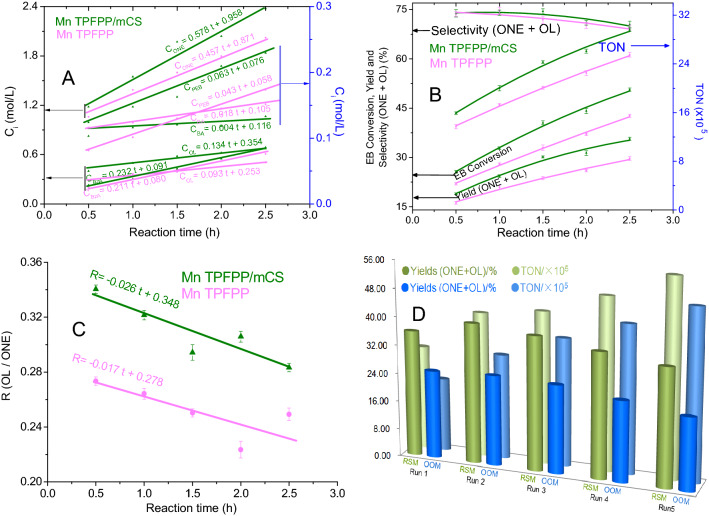
Catalytic activity and efficiency of Mn TPFPP/mCS and Mn TPFPP.

Therefore, the ethyl benzene oxidation catalyzed by Mn TPFPP/mCS, was conducted in the best oxidation reaction conditions above, the results are shown in Fig. 6.

### Catalysis of Mn TPFPP/mCS for ethyl benzene oxidation under the best catalytic reaction conditions

**Scheme 4:**
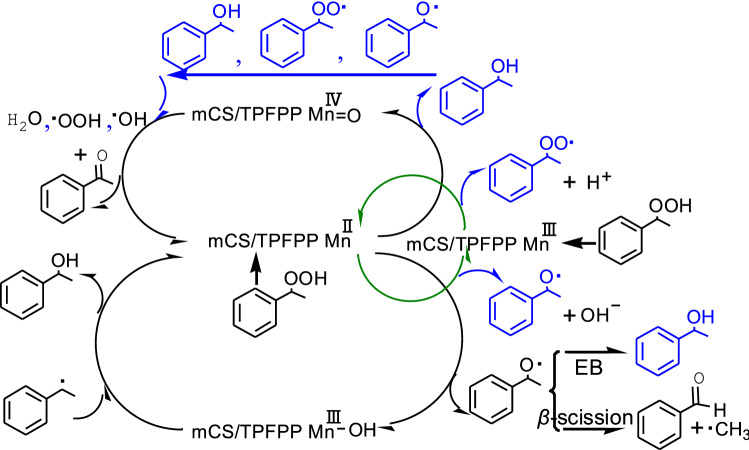
Possible mechanism for ethyl benzene oxidation over Mn TPFPP/mCS.

After the internal influencing factors on the catalytic performance of Mn TPFPP were well modulated by the way of coordinating and grafting on mCS. It would be promoting the enhancement of catalytic activity of Mn TPFPP/mCS. Then, under the RSM optimized oxidation reaction conditions, a 1st running experimental was conducted. The experimental results indicates that, both of Mn TPFPP/mCS and Mn TPFPP could catalyze ethyl benzene oxidation into acetophenone (ONE),1-phenylethanol (OL), benzaldehyde (BA), benzoic acid (BzA), and phenylethyl benzoate (PEB) (Fig. 6A). However, their catalytic activities were quite different. Because changes in concentration of each corresponding product got from ethyl benzene oxidation over the both catalysts with reaction times were different (Fig. 6A). Obviously, exception for benzaldehyde, the generation speeds of other four products obtained from ethyl benzene oxidation over Mn TPFPP/mCS were quicker than those obtained from Mn TPFPP. Compared with Mn TPFPP, the generation rate constants of OL, ONE, BzA, and PEB promoted by Mn TPFPP/mCS, were raised approximately 44%, 24%, 10%, and 47%, respectively. But the generation rate constant of BA decreased about 78%. In comparison, the selectivity to main products increased only a little (Fig. 6B). However, the comprehensive results were that, ethyl benzene conversion, TON and yields (ONE + OL) all increased, and the increment percents were 19%, 16%, and 20% respectively (Fig. 6B). In addition, Mn TPFPP/mCS could be reused more than four times, offering in average ethyl benzene conversion of 51.2%, TON of 4.37 × 10^6^, and yields (ONE + OL) of 36.4% (Fig. 6D). The reason resulted in the excellent catalysis is that: First, the specially coordination of NH_2_–C_sp_^3^ unit in mCS to the Mn ion of Mn TPFPP, increased the positive charges of the manganese ion. This greatly promoted the discomposing rate of 1-phenylethyl hydroperoxide into acetophenone (Scheme 4) and greatly decreased the ratio of OL/ONE (Fig. 6C). Because once 1-phenylethyl hydroperoxide was produced in ethyl benzene autoxidation^50^, which was immediately catalyzed and then discomposed by ${\text{mCS/TPFPP}}\;{\text{M}}\mathop {\text{n}}\limits^{{{{\rm III}}}}$ and ${\text{mCS/TPFPP}}\;{\text{M}}\mathop {\text{n}}\limits^{{{{\rm II}}}}$^51^ into 1-phenylethanol, 1-phenylethyl peroxyl radical, and 1-phenylethoxy radical. They were further catalytically oxidized by ${\text{mCS/TPFPP}}\;{\text{M}}\mathop {\text{n}}\limits^{{{{\rm IV}}}} = {\text{O}}$^52^ into acetophenone (Scheme 4). This resulted in that, the rate constant of generating acetophenone was more than 4 times as much as that of forming 1-phenylethanol (Fig. 6A), even if ${\text{mCS/TPFPP}}\;{\text{M}}\mathop {\text{n}}\limits^{{{{\rm III}}}} - {\text{OH}}$ could make hydroxylation of 1-phenylethyl radical into 1-phenylethanol (Scheme 4). The possible mechanism is proposed in Scheme 4 which is based on the following facts: The detected amount of 1-phenylethyl hydroperoxide using chemical analysis was very small (less than 0.08%), and the ethyl benzene oxidation was inhibited by butylated hydroxytoluene.

Second, when the amount of manganese element in 4th-reused Mn TPFPP/mCS catalyst was determined by ICP-OES, content of manganese species was almost not changed. The excellent stability of Mn TPFPP/mCS can be seen from Fig. S2 and S3: Compared Mn element of Mn TPFPP/mCS with that of Mn TPFPP/mCS-5th-resued, 9.1% (at%) of Mn were lost in five runs (Fig. S2). On average, in each run, Mn element was lost only 1.8% which was probably resulted from part amino groups oxidized by O_2_ and then lost. At the ranges of oxidation temperatures, Mn TPFPP/mCS mainly lost its water component (Fig. S3). The catalytic efficiency of Mn TPFPP/mCS was promoted so much, this is because the effects of grafting and coordinating action (internal factors) on the catalytic efficiency, were well modulated. That is to say, the stability of Mn TPFPP/mCS had been greatly improved.

Third, compared with the OOM optimized oxidation reaction conditions, under RSM optimized oxidation conditions (Fig. 6D), when the ethyl benzene oxidation time was at 2.5 h, in five runs, on average, Mn TPFPP/mCS could increase ethyl benzene conversion, TON and Yields (ONE + OL), 17%, 26% and 53%, respectively. The catalytic activity of Mn TPFPP/mCS was promoted so much, this is because the effects of external factors on the catalytic performance, were well tuned and controlled.

Reviewing and comparing the previous researches on hydrocarbon oxidation, although there were a lot of the influencing factors on the performances of catalysts and the interactions of the factors were complex^49^, it is worthy to use RSM optimizing the reaction conditions of ethyl benzene oxidation catalyzed by Mn TPFPP/mCS catalyst, including the our previous research works^34,35,53,54^, and maybe even some of other research works^1–25^. Because the method could tune the external factors (oxidation reaction conditions) to the optimal degree, probably letting the catalysts to make full use of the optimal external reaction conditions^55^.

In summary, compared with the present advanced technologies of ethyl benzene oxidation^1–6^, this catalytic oxidation of ethyl benzene offers an environmentally friendly technology.

## Conclusion

After utilizing the coordinating and grafting method to modulate the internal factors, which affected the catalytic performance of Mn TPFPP/mCS catalyst, and adopting Response Surface Methodology to optimize the external factors under environmentally friendly oxidation reaction conditions, the experimental results were obtained. These results indicate that, (1) among all type parameter terms, the single parameter (X_3_) and the quadratic parameter (X_3_^2^ and X_1_^2^) showed the most significant effect on the catalytic activity (TON). (2) Compared with the OOM-optimized oxidation reaction conditions, under the RSM-optimized oxidation reaction conditions (176.56 °C, 0.59 MPa, and 0.25 mg amount of Mn TPFPP), the catalyst material could be reused at least four times. The ethyl benzene conversion, the catalyst turnover numbers, and the yields increased 17%, 26% and 53% in average, reaching up to 51.2%, 4.37 × 10^6^ and 36.4%, respectively. (3) Relative to Mn TPFPP, Mn TPFPP/mCS could be reused more than four times, in average offering 51.2% ethyl benzene conversion, 4.37 × 10^6^ catalyst turnover number and 36.4% yields. Ethyl benzene conversion, catalyst turnover number and yields were respectively increased 19%, 16%, and 20%. This offers an environmentally friendly catalytic technology of ethyl benzene oxidation.

## Supplementary information

Supplementary information
