# Supplementary material for: Full use of factors promoting catalytic performance of chitosan supported manganese porphyrin
Source: Sci Rep. 2020 Aug 24;10:14132. doi: 10.1038/s41598-020-70210-y (PMC7445284; doi:10.1038/s41598-020-70210-y)
Supplement: Supplementary file 1 — Supplementary information [file 41598_2020_70210_MOESM1_ESM.docx]

Supplementary Material

**Full Use of Factors Promoting Catalytic Performance of Chitosan Supported Manganese Porphyrin**

Xian-Fei Huang ^a,^*, Gao-Cai Wang ^c^, Lin-Qiang Mo ^b^, Guan Huang ^b,^*, Peng Liu ^b^


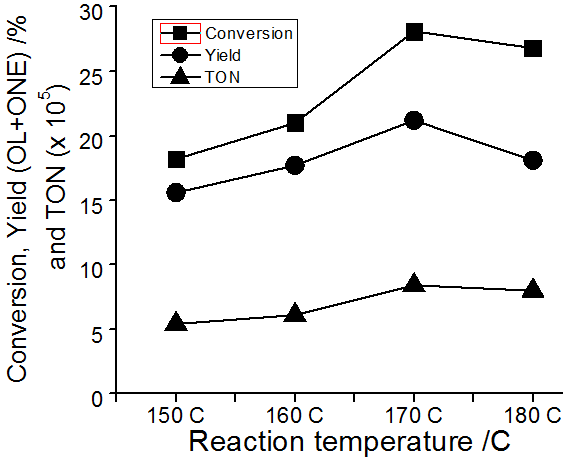


1.Fig.S1-1 Changes in the catalyst turnover number (TON), conversion and yields with oxidation temperature under ethylbenzene oxidation catalyzed by Mn TPFPP/np-CTS. Reaction conditions: 0.8 MPa, 0.50 mg Mn TPFPP, 2.5 h reaction time, 200 ml of ethylbenzene, 200 rpm of stirring speed, 0.03 m^3^/h of tail gas flow rate and no any solvents.


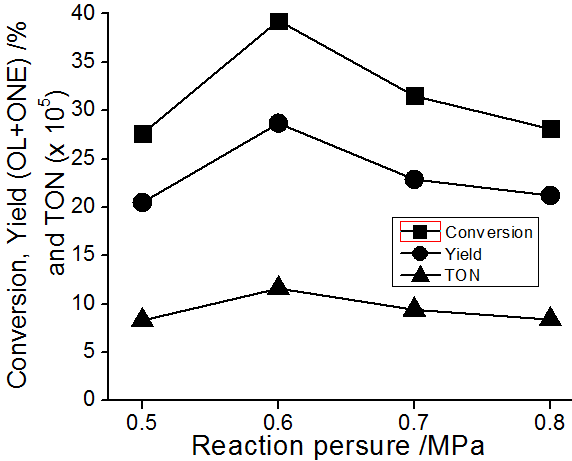


2.Fig.S1-2 Changes in the catalyst turnover number (TON), conversion and yields with oxygen pressure under ethylbenzene oxidation catalyzed by Mn TPFPP/np-CTS. Reaction conditions: 170°C, 0.50 mg Mn TPFPP, 2.5 h reaction time, 200 ml of ethylbenzene, 200 rpm of stirring speed, 0.03 m^3^/h of tail gas flow rate and no any solvents.


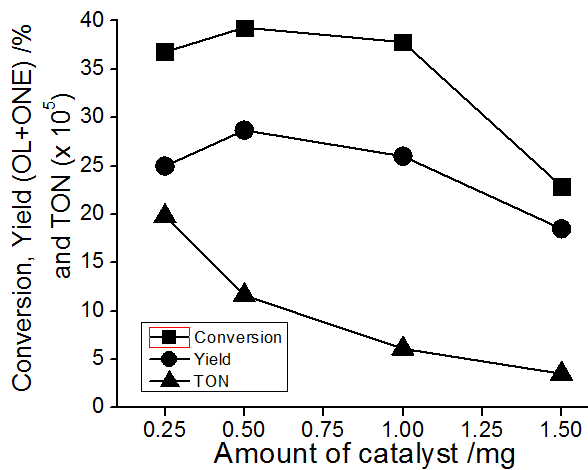


3.Fig.S1-3 Changes in the catalyst turnover number (TON), conversion and yields with amount of Mn porphyrin in ethylbenzene oxidation catalyzed by Mn TPFPP/np-CTS. Reaction conditions: 170°C, 0.6 MPa, 2.5 h reaction time, 200 ml of ethylbenzene, 200 rpm of stiring speed, 0.03 m^3^/h of tail gas flow rate and no any solvents.


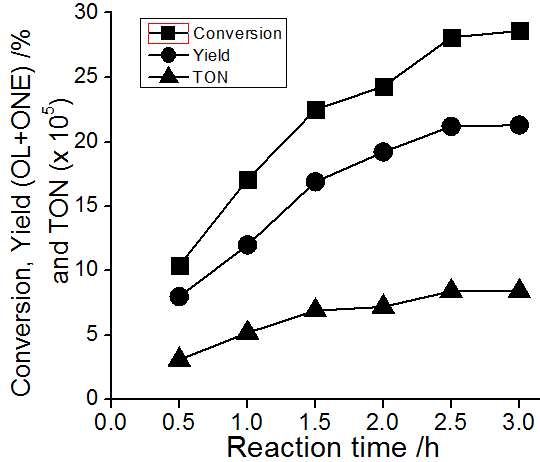


4.Fig.S1-4 Changes in the catalyst turnover number (TON), conversion and yields with reaction time in ethylbenzene oxidation catalyzed by Mn TPFPP/np-CTS. Reaction conditions: 170 °C, 0.8 MPa, 0.50 mg Mn TPFPP, 200 ml of ethylbenzene, 200 rpm of stirring speed, 0.03 m^3^/h of tail gas flow rate and no any solvents.

5. Energy dispersive spectra (EDS) characterization of Mn TPFPP/mCS and Mn TPFPP/mCS -5^th^-resued


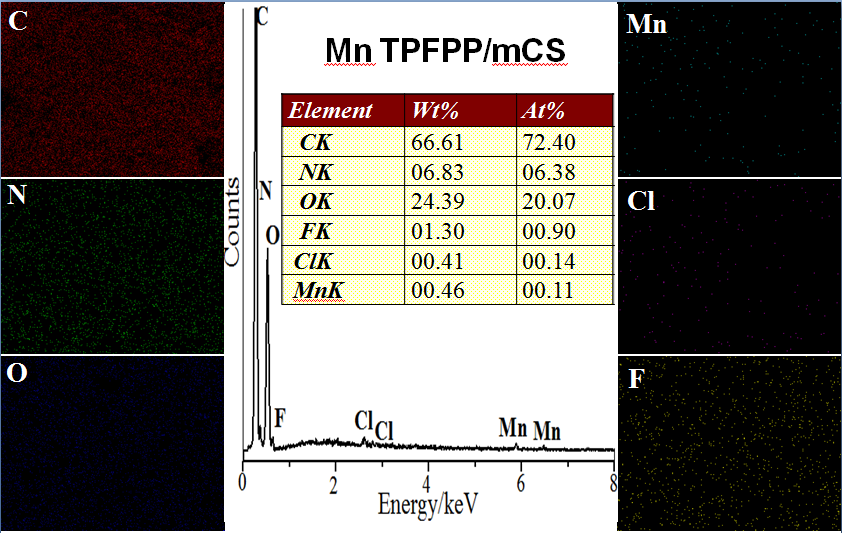

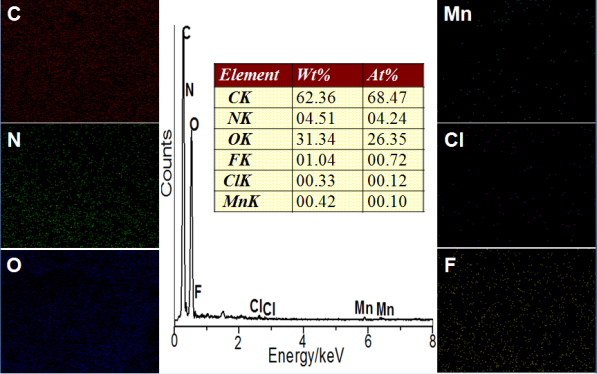


**Mn TPFPP/mCS (5^th^-recycle)**

Fig.S2 EDS images of Mn TPFPP/mCS and Mn TPFPP/mCS (5^th^-recycle),

inserts are the EDS elemental mapping of Mn, Cl, F, O, N and C elements.

6.Characterizaiton of thermogravimetric and differential scanning calorimetry of Mn TPFPP/mCS

Fig.S3 TG and Heat flux curves of Mn TPFPP/mCS

7.Elemental analysis of Mn TPFPP (C_44_H_8_F_20_N_4_MnCl), mCS, and Mn TPFPP/mCS, which were the starting materials:

Anal. Calcd for C_44_H_8_F_20_N_4_MnCl**:** C,49.68; H,0.75; F, 35.75; N,5.27; Mn, 5.17; Cl,3.34.

Found: C,49.64; H,0.72; F, 35.71; N,5.23; Mn, 5.14; Cl,3.30.

Anal. Calcd for mCS: hard to be conducted

Found: C,55.11; O,28.77;N,8.52;H,7.51

Anal. Calcd for C_44_H_8_F_20_N_4_MnCl/mCS: hard to be conducted

Found:C,49.70; O,35.74;N,7.65;H,6.76; F,0.016; Mn,0.0026;Cl,0.0015.
